# Report of the Committee on Hygiene of Paris, in View of the Approach of Cholera

**Published:** 1849-06

**Authors:** 


### SELECTIONS

FROM

### AMERICAN AND FOREIGN JOURNALS.

---

*Report of the Committee on Hygiene of Paris, in view of the approach of Cholera.*—The reappearance of cholera within the last year, both in this country and Europe, has called forth so many and such ably written reports touching the most efficacious hygienic measures to be adopted during the reign of the epidemic, that we have deemed it proper to omit the portion of the report of the Paris Hygiene Committee which relates to this subject, and translate only the section entitled—

*Conduct to be observed by persons attacked with Cholera before the arrival of Medical aid.*—Cholera is a non-contagious disease, not communicable by contact, and we may, consequently, afford, without fear, to persons laboring under the disease, the aid and attention which their situation requires.

It is to be desired that this opinion, the result of the most extended experience, acquired during the epidemic of 1832, and since, both in this country and other parts of Europe, where cholera has prevailed, may be made known to all classes of the people, because of the security which it affords the sick in dispelling a fear as unfortunate as groundless.

It should, however, be inculcated with equal force that, if experience has superabundantly proved that simple contact, and even constant association with individuals laboring under cholera, are incapable of developing the disease, it is not the less a fact that, during the prevalence of epidemics, the accumulation of the sick in narrow, damp, badly aired situations; in a word, in improper hygienic conditions, may greatly favor both the intensity of the disease and its extension into adjacent localities. The interests of the sick no less than the public weal require that persons suffering from cholera should, when occupying unhealthy habitations, be removed to more salubrious situations, since, under such circumstances, the aid afforded there will be more efficient and diminish the liability to the spread of the disease.

Experience teaches that, during epidemic cholera, derangements of the digestive functions prevail to a considerable extent; these derangements, ordinarily slight, do not constitute cholera, but may lead to it when neglected; hence it is of the greatest importance to prevent or correct them the moment they appear.

Every one attacked with pains in the stomach, colic, or diarrhea, should, before every thing else, and when even these symptoms seem to be of no gravity, pay great attention to the character of their food, diminish the quantity, or even abstain entirely, according to the urgency of the case; fatigue, cold, and humidity should be carefully avoided; the individual should go warmly clad, wrap the abdomen in a flannel jacket, with the view of preventing, as much as possible, this part of the body from becoming chilled, and drink some mild infusions of tea or of slightly aromatic plants—*sage*, *chamomile*, *garden balm*, *etc.*

Where the indisposition does not promptly disappear a physician should be sent for immediately.

It is very seldom that the attacks themselves of cholera are not ushered in by certain precursory symptoms; these are of the same nature as those just alluded to; they affect especially and primarily the digestive apparatus, that is to say, the stomach and bowels; and both these first symptoms and the disease itself are most easily, when most promptly, treated. In general, in this first period, the disease is not rebellious to well directed attentions; promptitude of succor is here the prime element of success, and, as this succor may be dispensed by any intelligent person, it is desirable that there should be constantly at the doors of the prisons, asylums, schools, in the poor and populous quarters of the city, a person, such as a sick nurse, or even a person a stranger, by profession, to waiting upon the sick, but intelligent, who would give the earliest attentions while awaiting the physician.

If the prescriptions, more hygienic than medical, indicated above, are not sufficient to arrest the derangement alluded to; if the diarrhea persists, the pain augments, and, especially if superadded to these there be vomiting, chilliness, coldness of the extremities; or if even these symptoms declare themselves abruptly without any precursory sign, as is the case with certain individuals, the proper course to pursue is to put the patient into a warm bed immediately, between blankets; to apply hot bricks, or bags of hot sand, or bottles of hot water to his feet, and warm napkins to the abdomen and stomach; to rub the extremities with flannel impregnated with some stimulating substance, as alcohol, brandy, oil, or spirits of camphor; to administer, at intervals of thirty minutes, warm drinks, gently tonic or aromatic, such as infusions of tea or of chamomile; to recall warmth to the extremities by means of cataplasms of ground flaxseed sprinkled with a small quantity of mustard; to avoid all cause of chilliness; to give very small injections of rice water, starch, or decoction of marsh mallow, to which should be added a decoction of poppy heads; it is better, if the patient is unable to retain it, to give a second, or even a third, than to give at one time a full injection, which would be with difficulty borne.

When to the preceding symptoms are joined pains in the head, cramps in the limbs, the persistence or extension of cold over a large portion of the body; if the tongue becomes cold, the eyes sunken and surrounded by a dark circle, the skin bluish upon the face and hands—these indications of great gravity in the disease should not cause the neglect of the means which we have pointed out, but, on the contrary, should be a reason for applying them with greater energy and perseverance until the physician, who should be expeditious shall arrive. Those who are first at the bedside should not be discouraged if the use of the means we have pointed out do not appear to effect any decided amelioration in the condition of the sick. The object which they should have in view is, to restore warmth to the patient, to re-establish the circulation and movements of the heart; and this result is not ordinarily attained until after the lapse of a considerable length of time. It is, therefore, indispensable to persevere uninterruptedly in the employment of these means, until we bring about the return of the natural heat of the body, which indicates a reaction that, as a general rule, is favorable.

It is in this period especially that it is of the greatest importance to confide the patient to the hands of a physician; the indications to be fulfilled being, from this moment, no longer understood by any other than the medical man, it would be useless and even dangerous to give for this stage of the disease instructions which would not be comprehended and which might be misinterpreted.

*Committee.*—Magendie, president; Aubert Roche, Royer Collard, &c., &c. D. W. Y.

